# St. George's Hospital and the Wounded

**Published:** 1915-03-06

**Authors:** 


### ST. GEORGE'S HOSPITAL AND THE WOUNDED.

At the outset of the war the house committee placed at the disposal of the Services 100 beds, part of which were at the convalescent hospital at Wimbledon, and on this offer being accepted fifty beds were at once prepared and kept open for the reception of sick and wounded soldiers.

This was necessary, as it was impossible for the authorities to inform the hospital's authorities when the beds would be called for and how many would be required at a time, but it at once raised a problem of some importance, as the grants from the big Funds are dependent upon the cost per bed, and the keeping open of a number of beds largely increases this cost.

In this respect the hospital experience is felt to be rather unfortunate, as it was some weeks before it received the first batch of wounded. From this time on, too, the number of beds was not kept up continuously, so that the average number of beds occupied during the year compares unfavourably with that during the previous year. This is an important point, as the house committee are making every effort to keep down the cost per bed, with due regard to the welfare of the patients.

As regards the arrangements for the treatment of the soldiers, it was clearly necessary that special regulations should be made for them, and that they could not be placed under the ordinary hospital restrictions. Many of them under ordinary circumstances would be treated as out-patients, their injuries and ailments not being sufficiently severe to necessitate admission into hospital or confinement to bed. Strong and healthy men, too, require more food than the average hospital patient, and therefore the ordinary diet had to be added to. Also it was naturally felt that, as far as possible, everything should be done to add to their happiness by enabling them to see their relatives and friends daily, allowing them to smoke, to go out for motor-rides, providing occasional entertainments, and so on. All this made it necessary that the soldiers should be placed in wards that could be cut off and completely separated from the rest of the hospital.

The arrangements made have given every satisfaction. A great deal of the additional expense involved has been met by generous donations of food made by our Colonies and by many benefactors; and although there has been no commissioned officer in residence, there has not been the slightest difficulty with the men, who have behaved extremely well.

With regard to entertainments outside the hospital, which have been arranged in great numbers by the charitable public, the hospital has found it necessary to make a regulation that the men shall be driven there and back under suitable guardianship, as otherwise, owing to the hospitality of another portion of the public, the time of their return and condition on return become doubtful.

Some trouble is caused by the apparently unnecessary secretarial work involved by the incessant correspondence and the large number of constantly changing forms and returns required by the War Office.

With regard to the character of the ailments, at first the wounded formed 75 per cent. of the whole, at the present time they form 30 per cent., the admissions from sickness (including many cases of frostbite) having largely increased of late. Antitetanic serum has been largely used, and the hospital has had no cases of tetanus, but experience at St. George's tallies with that of other hospitals in that all the bullet and shrapnel wounds have been septic on arrival. The hospital's experience, too, agrees with the figures mentioned in the House of Commons, that approximately 60 per cent. of the wounded are able to return to the Front.

It is satisfactory to be able to report that the interests of the district served by the hospital have hardly suffered, as all urgent cases have been able to be admitted as usual, but the hospital has been obliged to refuse to admit many cases from a distance.

